# Supplementary material for: TMBIM4 Deficiency Facilitates NLRP3 Inflammasome Activation-Induced Pyroptosis of Trophoblasts: A Potential Pathogenesis of Preeclampsia
Source: Biology (Basel). 2023 Jan 29;12(2):208. doi: 10.3390/biology12020208 (PMC9953300; doi:10.3390/biology12020208)
Supplement: Supplementary file 1 [file biology-12-00208-s001.zip › Figure S1.pdf]

Figure-2I

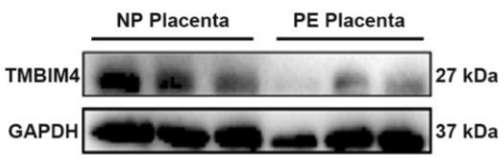

TMBIM4

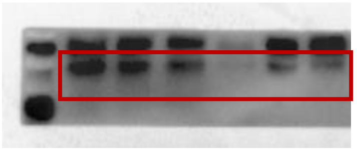

GAPDH

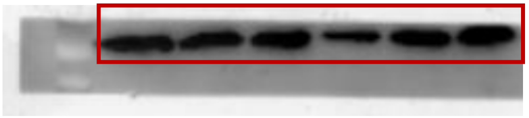

Figure-4L

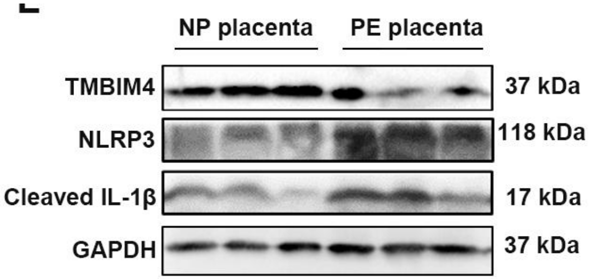

TMBIM4

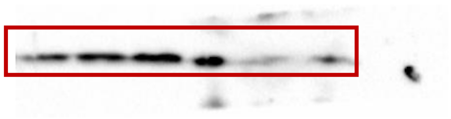

IL-1β

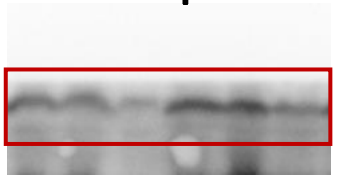

NLRP3

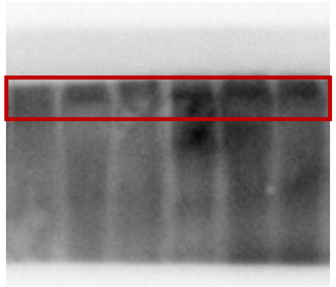

GAPDH

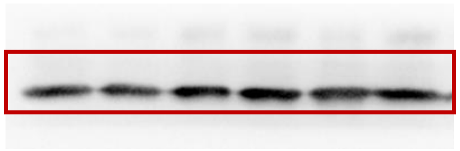

Figure-3F

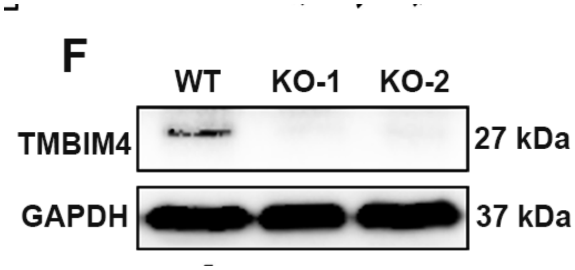

TMBIM4

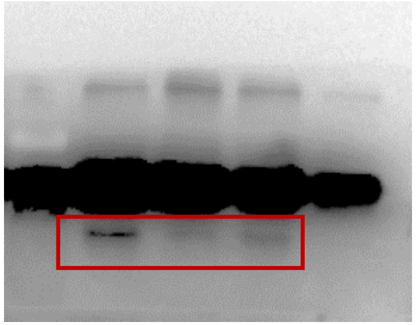

GAPDH

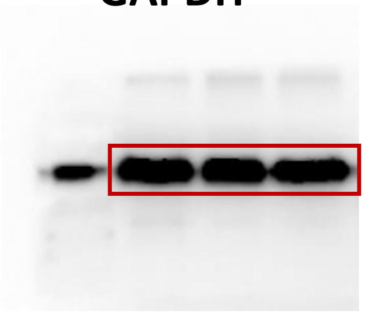

Figure-3A

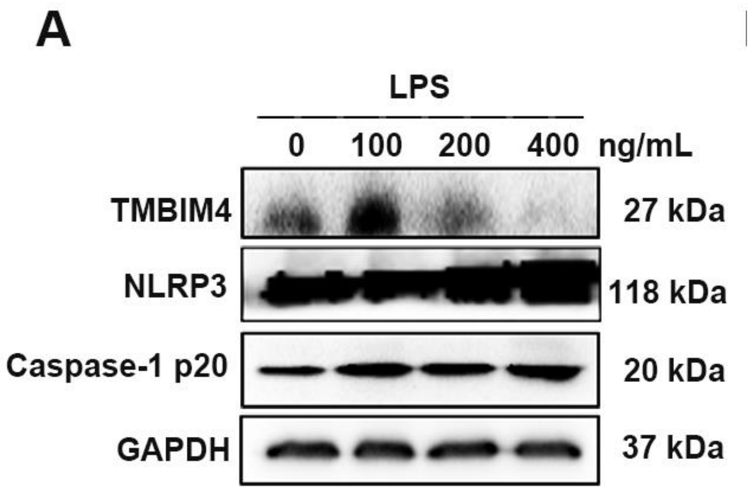
A
LPS
0
100
200
400
ng/mL
TMBIM4
NLRP3
Caspase-1 p20
GAPDH
27 kDa
118 kDa
20 kDa
37 kDa

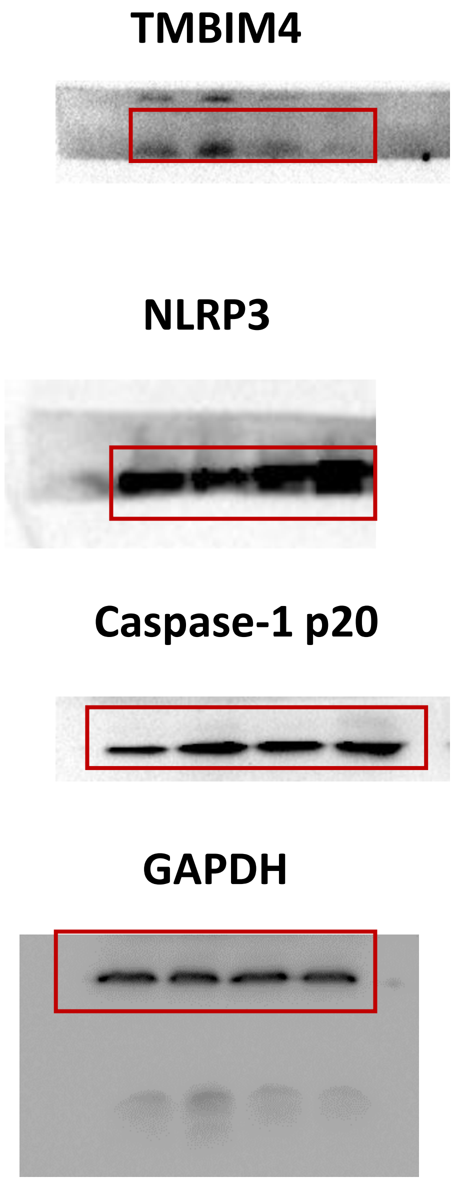
TMBIM4
NLRP3
Caspase-1 p20
GAPDH

Figure-4A

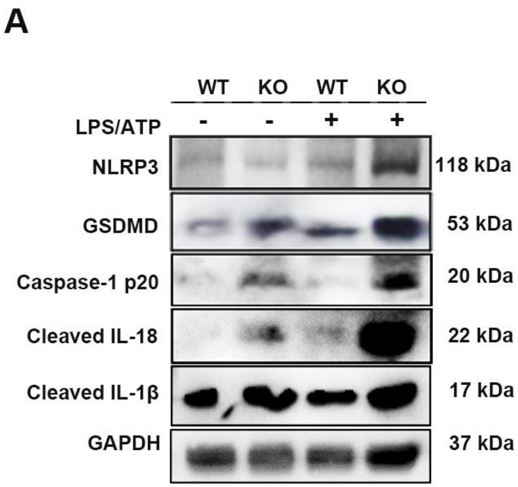

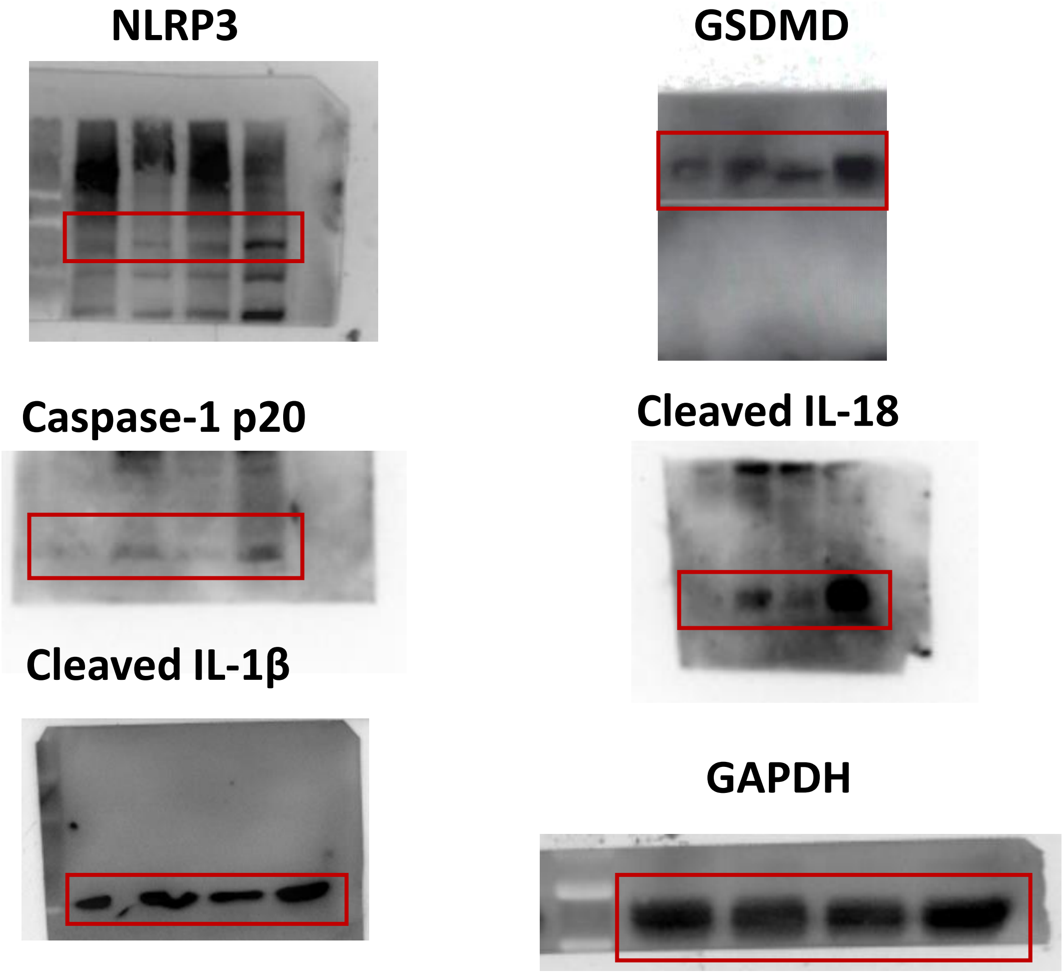

Figure S1. Contains images of the original blots.
