# Supplementary material for: TMBIM4 Deficiency Facilitates NLRP3 Inflammasome Activation-Induced Pyroptosis of Trophoblasts: A Potential Pathogenesis of Preeclampsia
Source: Biology (Basel). 2023 Jan 29;12(2):208. doi: 10.3390/biology12020208 (PMC9953300; doi:10.3390/biology12020208)
Supplement: Supplementary file 1 [file biology-12-00208-s001.zip › Table S1.pdf]

Table S1. Characteristics of women with early-onset PE and gestationally matched controls

Clinical data	NP (n=3)	PE (n=3)	*P* -value
Age (years)	30.0±2.8	30.3±2.4	0.9043
Gestational age at delivery (weeks)	38.0±0.8	37.7±0.5	0.6433
MABP (mmHg)	84.0±9.9	105.4±3.7	0.0457
BMI (kg/m²)	22.9±2.0	23.1±1.9	0.9108
Neonatal weight (g)	3193.3±180.0	2656.6±74.1	0.0175
Proteinuria	None	++	N/A

***BMI*, body mass index; *MABP*, mean the average arterial blood pressure; *NP*, Normal pregnancy; *PE*, preeclampsia**
